# Long-term clinical and echocardiographic outcomes of extensive septal myectomy for hypertrophic obstructive cardiomyopathy in Chinese patients

**DOI:** 10.1186/s12947-016-0060-9

**Published:** 2016-05-17

**Authors:** Lei Yao, Li Li, Xiong-Jun Lu, Yan-Ling Miao, Xiao-Ning Kang, Fu-Jian Duan

**Affiliations:** 1 Second Division of Ultrasound Diagnosis, Cangzhou Central Hospital, 16 Xin-Hua-West Street, Cangzhou, Hebei 061001 People’s Republic of China; 2 Cardiothoracic Surgical Department, Shandong Chinese Medical Hospital, Jinan, 250011 China; 3 Ultrasonic Imaging Center, Fuwai Hospital, Chinese Academy of Medical Sciences, Beijing, 100037 China

**Keywords:** Hypertrophic cardiomyopathy, Hypertrophic obstructive cardiomyopathy, Extensive septal myectomy

## Abstract

**Background:**

There has been limited data addressing outcomes of extensive septal myectomy in Chinese patients with hypertrophic obstructive cardiomyopathy (HOCM). In this study, the objective was to evaluate the clinical and echocardiographic outcomes of extensive septal myectomy in a relative large number of Chinese HOCM patients over long-term follow-up.

**Methods:**

We retrospectively studied 139 consecutive HOCM patients (age 43 ± 15 years, 37 % male) who underwent extensive left ventricular septal myectomy. During the perioperative period, all patients were examined by echocardiography. All-cause death and cardiac death were considered as primary endpoints during follow-up. Perioperative data was obtained by retrospective review of institutional surgical databases. Follow-up data of echocardiography and clinical status was recorded through outpatient interview.

**Results:**

Perioperative events consisted of arrhythmia, retraction injury to aortic valve leaflets, pleural effusion, and hemodialysis and the use of intra-aortic balloon pump. There was no in-hospital mortality. The follow-up period averaged 5.6 ± 0.9 years and overall survivals were 100.0, 99.3, 99.3, 98.5 and 97.8 % at 1, 2, 3, 4 and 5 years, respectively. Left ventricular outflow tract (LVOT) gradient decreased form preoperative 84 ± 17 mmHg to 12 ± 3 mmHg at 2.5 years after surgery and it further reduced to 6 ± 3 mmHg at 5 years after surgery (*P* < 0.05). Compared with the preoperative levels, interventricualr septal thickness decreased by 32 % while diastole left ventricular inner diameter approximately increased by 10 % and ejection fraction (EF) was significantly elevated during follow-up (*P* < 0.05). By echocardiography detection, mitral regurgitation was ameliorated for HOCM patients after surgery. There was significant improvement in New York Heart Association (NYHA) class. The proportion of NYHA III and IV decreased from preoperative 58 to 19 % at 2.5 years after surgery and it reduced to 11 % at 5 years after operation.

**Conclusion:**

Extensive septal myectomy offers minimal operative risk and provides long-term relief for LVOT obstruction in Chinese HOCM patients.

## Background

Hypertrophic cardiomyopathy (HCM) is estimated to affect 0.2 % of the population [1, 2]. As the most common inherited cardiac disease, HCM is characterized by asymmetrical septal hypertrophy [3, 4]. The phenotypes are ranging from minor to severe life-threatening status [5]. Left ventricular outflow tract (LVOT) obstruction is responsible for disabling symptoms in a large proportion of patients and confers a worse prognosis. HCM is the most common cause of sudden cardiac death (SCD) in people aged less than 35 years. In fact, it is more common for HCM patients to develop to severe progressive heart failure than SCD.

Septal myectomy is considered as the gold standard for septal reduction therapy in patients with dynamic LVOT obstruction [6]. The long-term mortality for hypertrophic obstructive cardiomyopathy (HOCM) patients with septal myectomy reduction therapy decreases to 1.4–1.8 % [7, 8]. Septal myectomy has contributed to the overall reduction in mortality and provided survival equivalent to that of the general population [9]. The operative technique of HOCM has undergone evolution from classic Morrow surgery to extensive resection. Extensive septal myectomy surgery has evolved into a comprehensive repair technique for LVOT obstruction and abnormality of the mitral apparatus [10]. Extension of the myectomy area leads to improved surgical effect. Currently, there are limited data addressing long-term outcome of extensive septal myectomy in Chinese patients with HOCM. In the present study, we sought to report the early and late clinical and echocardiographic outcomes of Chinese HOCM patients who underwent extensive septal myectomy surgery.

## Methods

### HOCM patients

This study was carried out in 139 consecutive HOCM patients treated with extensive surgical myectomy in Cangzhou Central hospital and Shandong Chinese Medical Hospital from Jan 1, 2008 to May 31, 2011. Patients were eligible for inclusion in surgical intervention if they met the following two criteria [11]: 1) LVOT pressure gradient ≥50 mmHg at rest or with physiological provocation by transthoracic echocardiography; 2) presence of severe symptoms despite prior appropriate medical therapy with beta-receptor blocker and calcium channel blocker. All patients were evaluated by consultant cardiologists. Although they were given medical therapy, they still had severe symptoms attributable to LVOT obstruction. The review of hospital records and analyses of preoperative data, operative reports, postoperative and follow-up echocardiography were carried out. In this study, perioperative period was defined as time period from surgical preparation to 30 days after extensive septal myectomy. The study had full approval from the Ethics Boards of Cangzhou Central hospital and Shandong Chinese Medical Hospital.

### Surgical procedure

Operations were carried out under mild hypothermic cardiopulmonary bypass with total anesthesia as previously described [12, 13]. The heart and ascending aorta were exposed by a longitudinal median incision in sternum. Cannulas were inserted into superior vena cava, inferior vena cava and ascending aorta to establish cardiopulmonary bypass. Through a transverse aortotomy, the aortic valve was exposed and cardiac asystole was induced by cold blood antegrade cardioplegia. Hypertrophic septum and mitral valve were exposed sufficiently by pulling right coronary aortic valve. A Ross retractor was used to display the muscular septum. The superior borderline of septal resection located at 3 mm below right coronary sinus valve. Resection started from middle of right aortic sinus and moved 10–12 mm horizontally toward commissure of left sinus valve and right sinus valve. Longitudinal resection usually reached the root of mitral papillary muscle, length of which ranged from 45 to 50 mm. In order to reduce LVOT gradient, the thicknesses of the left ventricular wall and interventricular septum need to be nearly normal by visual inspection.

The following extended procedures were performed as previously described [14, 15]: 1) The resection was continued toward the mitral valve annulus and apically to the bases of the papillary muscles. 2) All areas of papillary muscle fusion to the septum or ventricular free wall are divided, and anomalous chordal structures and fibrous attachments of the mitral leaflets to the ventricular septum are excised. 3) Plication of the anterior mitral leaflet (AML) was performed if there were indications by selection criteria including floppy and lax anterior leaflet, AML ≥ 3.0 cm, mitral regurgitation ≥ 2+, systolic anterior motion (SAM), and/or absence of rheumatic or other intrinsic mitral valve disease. Intraoperative transesophageal echocardiography (TEE) was used routinely. TEE assessment was performed after weaning from cardiopulmonary bypass to evaluate adequacy of LVOT and mitral valve function.

Postoperative monitor was carried out to maintain proper cardiac preload and appropriate colloid infusion. Beta-receptor blocker or calcium-channel blocker was applied routinely. Low-dose vasoactive drugs were used when hemodynamic circulation fluctuated.

### Echocardiography

During hospital stay, preoperative transthoracic echocardiography was performed for HOCM patients and intraoperative TEE was applied routinely. The adequacy of the resection and the LVOT gradient were assessed immediately by transthoracic echocardiography after surgery and transthoracic echocardiography was repeated before or on the day of hospital discharge. During outpatient follow-up period, patients had transthoracic echocardiography detection at every visit. A careful analysis of septal hypertrophy, abnormalities of the mitral valve, and subvalvular apparatus was performed. LVOT gradient was detected at rest and with provocation by transthoracic echocardiography. Specific details of mitral valve length, SAM, and mitral regurgitation were evaluated. Images were captured on cine loops at the time of the detection. M-mode from long axis view was used to measure interventricualr septal (IVS) thickness and diastole left ventricular inner diameter (LVID_d_). M-mode from short axis view was used to measure thickness of left ventricular wall. Ejection fraction (EF) was automatically calculated by the measurement package.

### Follow-up

The follow-up period averaged 5.6 ± 0.9 years for HOCM patients. Follow-up data were derived from medical charts and LVOT gradient information was obtained from the record of transthoracic echocardiography detection at every outpatient visit. Our primary endpoints included all-cause death and cardiac death during follow-up. Cardiac death was defined as a death resulting from heart failure or SCD. SCD was defined as an abrupt loss of consciousness within 1 h after the onset of acute symptoms, and the cause of death could not be attributed in the postmortem examination. All patients were assigned New York Heart Association (NYHA) classification based on symptoms.

### Statistical analysis

Continuous variables were expressed as mean ± SD otherwise described as proportion. *χ* ^2^ test was used to compare categorical variables. For continuous variables, one-way ANOVA was used to analyze the data among three groups and then paired *t* test was performed for the comparison between two groups. The Kaplan-Meier method was used to draw survival curve and calculate survival rate and cumulate hazard. Difference was considered statistically significant when *P* < 0.05. All statistical tests were performed using SPSS software package 19.0 for Windows.

## Results

### Clinical characteristics

Clinical characteristics of 139 HOCM patients with mean age 43 ± 15 years were shown in Table 1. Females were more than males. Body mass index was 28 ± 8 kg/m^2^ and heart rate was 74 ± 5 beat/min. All patients received medical therapy with beta-receptor blocker or calcium channel blocker. Most patients had severe preoperative symptoms including dyspnea, chest pain, and syncope. Family history of HCM was presented in 54.7 % of subjects. Hypertension, diabetes and dyslipidemia were prevalent in the HOCM patients. The majority of HOCM patients belonged to NYHA III.

**Table 1:** Clinical characteristics

Variables	Values
*n*	139
Age (y)	43 ± 15
Male	52 (37.4 %)
Body mass index (kg/m^2^)	28 ± 8
Heart rate (beat/min)	74 ± 5
SBP (mmHg)	113 ± 12
DBP (mmHg)	73 ± 9
Symptoms	
Dyspnea	133 (95.7 %)
Chest pain	73 (52.5 %)
Syncope	33 (23.7 %)
Hypertension	44 (31.7 %)
Diabetes	25 (18 %)
Dyslipidemia	101 (72.7 %)
Family history of HCM	76 (54.7 %)
Medical therapy	
Beta-receptor blocker	16 (11.5 %)
Calcium channel blocker	43 (30.9 %)

*SBP* systolic blood pressure, *DBP* diastolic blood pressure

### Perioperative events and complications

Adjunctive surgical procedures were summarized in Table 2. Overall, cardiopulmonary bypass time was 133 ± 40 min and aorta cross-clamp time was 85 ± 26 min. Postoperative intensive care unit (ICU) stay time was 3 ± 3 days. Mechanical ventilation time was 24 ± 15 h. Postoperative hospital stay time was 10 ± 5 days.

**Table 2:** Adjunctive procedures

Procedure	*n* (%)	Age (y)
CABG	11 (7.9 %)	56 ± 16
LV aneurysmectomy	2 (1.4 %)	40 ± 17
MV replaement	7 (5 %)	46 ± 14
MV repair	17 (12.2 %)	44 ± 9
AV repair	4 (2.9 %)	61 ± 19
TV repair	6 (4.3 %)	42 ± 16

*CABG* coronary artery bypass graft, *LV* left ventricle, *MV* mitral valve, *AV* aortic valve, *TV* tricuspid valve

The 139 patients had no early death within 30 days after extensive septal myectomy. Perioperative arrhythmia was shown in Table 3. In this study, perioperative arrhythmia events included atrial fibrillation, atrial premature beat, ventricular premature beat, atrioventricular block, left bundle branch block, left anterior fascicular block and right bundle branch block. There was a preoperative history of atrial fibrillation in 13 patients while postoperative new atrial fibrillation occurred in 11 patients. In total, there were 16 patients suffering from atrial ventricular block after surgery. Permanent pacemaker was implanted for 8 (5.8 %) patients with complete atrioventricular block. None of the patients needed implantable cardioverter defibrillator (ICD) during early postoperative period. In addition, retraction injury to aortic valve leaflets occurred in 5 patients. One patient needed the mechanical support of intra-aortic balloon pump (IABP) immediately following surgery. Postoperative pleural effusion happened in 25 patients. Hemodialysis was used for 2 patients. Two patients were subjected to second intubation and 1 patient underwent tracheotomy. Second transfer to ICU was required for 1 patient on postoperative 7th day.

**Table 3:** Postoperative arrhythmia

Arrhythmia	Number	Percentage
Atrial fibrillation	24	17.3 %
Atrial premature beat	7	5.0 %
Ventricular premature beat	4	2.9 %
Atrioventricular block	16	11.5 %
Left bundle branch block	34	24.5 %
Left anterior fascicular block	10	7.2 %
Right bundle branch block	5	3.6 %

### Clinical and echocardiographic follow-up

**Fig. 1:**
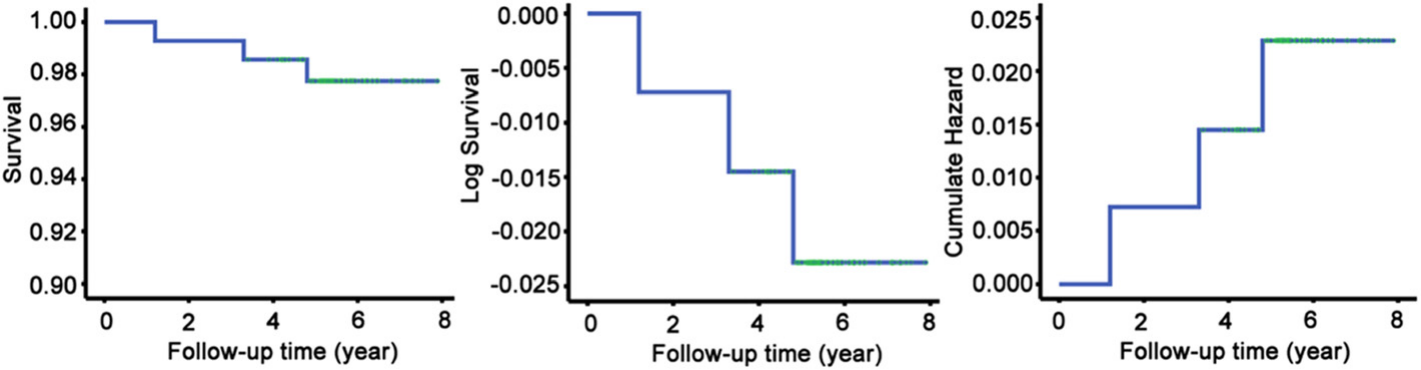
Kaplan-Meier curve for HOCM patients during follow-up period. Survival rate and log survival rate decreased whereas cumulate hazard gradually increased during follow-up time. The survival rate was 100 % at the first year after surgery and then decreased to 99.3 % at the second and the third year. The survival rate was 98.5 % at the fourth year and reduced to 97.8 % at the fifth year

Clinical follow-up was 5.6 ± 0.9 years (minimum 1.2 years and maximum 7.9 years). Four patients were lost to late follow-up. Overall survival was 100.0, 99.3, 99.3, 98.5 and 97.8 % at 1, 2, 3, 4 and 5 years, respectively (Fig. 1). One patient died of cardiac origin and 2 patients had undiagnosed sudden death.

**Fig. 2:**
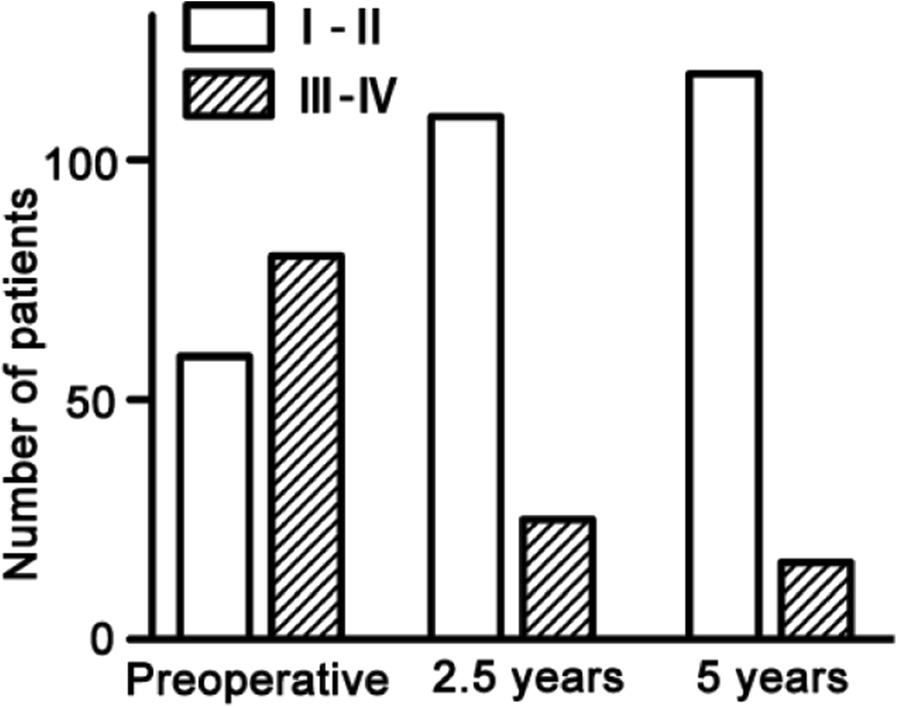
Improvement in cardiac function after extensive septal myectomy. The numbers of patients in NYHA Class I-II and NYHA Class III-IV were calculated before surgery, 2.5 years and 5 years after surgery, respectively. *χ* ^2^ test was performed to analyze the differences among groups. There was significant difference in preoperative and postoperative cardiac function (*P* < 0.05). There was no difference in cardiac functions between 2.5 years after surgery and 5 years after surgery (*P* > 0.05). *n* = 59 for NYHA Class I-II and *n* = 80 for NYHA Class III-IV before surgery; *n* = 110 for NYHA Class I-II and *n* = 25 for NYHA Class III-IV at 2.5 years after surgery; *n* = 117 for NYHA Class I-II and *n* = 15 for NYHA Class III-IVat 5 years after surgery

**Fig. 3:**
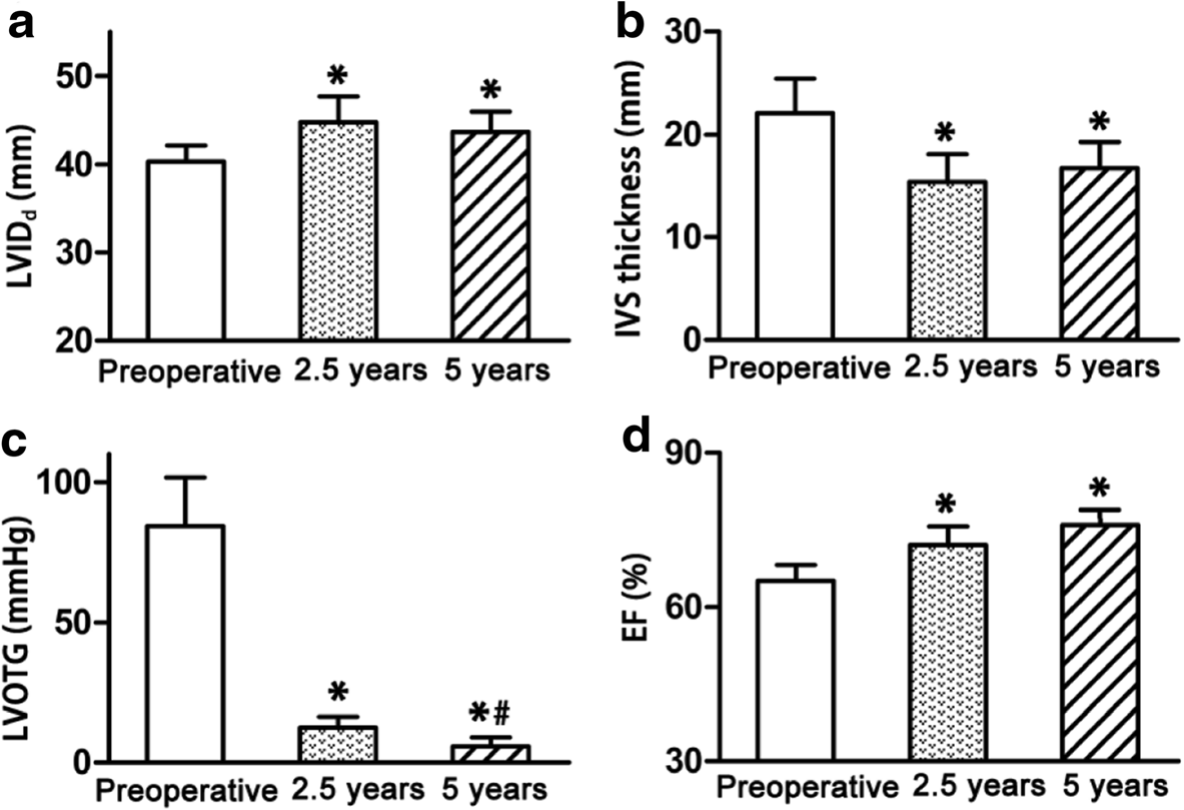
Changes in echocardiographic parameters during follow-up period. **a**. Changes in LVID_d_ during follow-up. **b**. Changes in IVS thickness during follow-up. **c**. Changes in LVOTG during follow-up. There was significant decrease in LVOTG at 2.5 years and 5 years after surgery. **d**. Changes in EF during follow-up. EF was elevated at 2.5 years and 5 years after extensive septal myectomy. LVID_d_: diastole left ventricular inner diameter; IVS: interventricular septal; LVOTG: left ventricular out tract gradient; EF: ejection fraction. ^*^ *P* < 0.05 vs preoperative group, ^#^ *P* < 0.05 vs 2.5 years group

Follow-up outcomes indicated the symptoms were significantly attenuated and physical abilities were increased. Improvement in NYHA class was shown in Fig. 2. The proportion of NYHA III and IV decreased from preoperative 58 to 19 % at 2.5 years after surgery while it reduced to 11 % at 5 years after operation. LVOT gradient decreased form preoperative 84 ± 17 mmHg to 12 ± 3 mmHg at 2.5 years after surgery and it further reduced to 6 ± 3 mmHg at 5 years after surgery (*P* < 0.05, Fig. 3). Compared with the level of preoperative IVS thickness, IVS thickness decreased by 32 % at postoperative 2.5 years and maintained the same low level at postoperative 5 years (*P* < 0.05, Fig. 3). LVID_d_approximately increased by 10 % at postoperative 2.5 years and 5 years, compared with the level before surgery (*P* < 0.05, Fig. 3). Similarly, EF was significantly elevated at 2.5 years and 5 years after extensive septal myectomy (*P* < 0.05, Fig. 3). By transthoracic echocardiography detection, mitral regurgitation was ameliorated for patients after surgery. There were 52 % of patients showing moderate mitral regurgitation and 8 % of patients had severe mitral regurgitation before surgery. Preoperative mild mitral regurgitation existed in 40 % of patients. With extensive septal myectomy, both severe mitral regurgitation and moderate mitral regurgitation disappeared. The proportion of postoperative mild mitral regurgitation increased to 89 and 11 % of patients did not have mitral regurgitation any more.

## Discussion

The present study included a relative large of Chinese HOCM patients who were treated by extensive septal myectomy in two hospitals. Our results showed that extensive septal myectomy significantly reduced LVOT obstruction, mitral regurgitation and HOCM-related symptoms. Cardiac function was obviously increased by surgical treatment. Extensive septal myectomy and adjunctive procedures could be an efficacious and low-risk therapy in Chinese HOCM patients. It gives predictable, immediate, and durable remodeling of the LVOT which translates into long-term control of symptoms.

### Extensive septal myectomy surgery in HOCM

HOCM, as a common type of hypertrophic cardiomyopathy, is mainly characterized by asymmetric septal hypertrophy, LVOT obstruction, diastolic dysfunction, cardiac ischemia as well as arrhythmia [16–18]. The aim of medical therapy is to abolish the catecholamine-induced effects that may exacerbate LVOT obstruction and to decrease heart rate which allows longer time for diastolic filling [17, 19, 20]. However, the early improvement for HOCM patients is often followed by clinical symptomatic impairments after conservative management with beta-blocker and/or calcium antagonist [21]. With septal myectomy reduction therapy, the long-term mortality for HOCM patients could significantly decrease to 1.4–1.8 % [7, 8].

Left ventricular septal myectomy was firstly reported by Cleland in 1963 and Morrow subsequently revealed the good clinical and hemodynamic outcomes of myectomy surgery [22]. Isolated septal myectomy mainly resected the bulge part of the hypertrophic septum. Some intraventricular anomalies such as mitral apparatus-related anomalies existed in HOCM patients. Under the condition, isolated septal myectomy could not diminish SAM of mitral valve and relieve LVOT obstruction completely [10]. Marwick et al. reported that up to 20 % of patients with isolated septal myectomy were placed back on cardiopulmonary bypass because of inadequate resection [23]. An inadequate length of septal excision was the most common reason of recurrent LVOT obstruction after myectomy [24]. Extensive septal myectomy to the midventricular level, with or without shaving of the papillary muscles, could eliminate the LVOT gradient and SAM-induced mitral regurgitation [25]. Knyshov G et al. performed a cohort study in HOCM patients and their results showed that LVOT gradient of HOCM patients reduced from 113.3 ± 14.9 mmHg to 17.3 ± 10.2 mmHg after extensive septal myectomy surgery [26].

### Follow-up outcomes of extensive septal myectomy surgery

Until now, there are limited data addressing long-term outcomes of extensive septal myectomy in Chinese HOCM patients. Wang et al. showed that extensive septal myectomy provided excellent relief from LVOT obstruction (91.8 ± 25.1 to 14.3 ± 13.4 mmHg, *P* < 0.05) and satisfactory clinical outcomes for 93 HOCM patients at early and mid-term follow-up [27]. Their study showed that the 30-day and in-hospital mortality was 0 % [27]. After surgery, limiting symptoms were decreased while physical abilities were increased [27]. In our study, the resection size was extended to release adhesion of mitral papillary muscle with left ventricular wall and satisfactory operative effects and clinical outcomes were obtained. The symptoms of HOCM patients undergoing extensive septal myectomy procedure in our study were obviously mitigated after operation. Echocardiography results by our study showed LVOT gradient and septal width of HOCM patients were significantly reduced whereas their LVID_d,_ and EF were increased after surgery. The most common postoperative complication was arrhythmia, which mainly consisted of left bundle branch block, atrial fibrillation, atrioventricular block and so on. In current study, the types and prevalence of perioperative arrhythmia were similar to the previous reports [28–30].

## Conclusion

Extensive septal myectomy and adjunctive procedures provide excellent relief of symptoms and improve cardiac function in Chinese HOCM patients with minimal surgical risk. Further studies with larger number and longer follow-up were expected to aim at examining the clinical outcomes of extensive septal myectomy surgery.

### Ethics approval and consent to participate

The authors stated that the study had full approval from the Ethics Boards of Cangzhou Central hospital and Shandong Chinese Medical Hospital.

## Abbreviations

AML: anterior mitral leaflet
EF: ejection fraction
HCM: hypertrophic cardiomyopathy
HOCM: hypertrophic obstructive cardiomyopathy
ICU: intensive care unit
IVS: interventricular septal
LVID_d_: diastole left ventricular inner diameter
LVOT: left ventricular outflow tract
NYHA: New York Heart Association
SAM: systolic anterior motion
SCD: sudden cardiac death
TEE: transesophageal echocardiography
